# Working memory training: from metaphors to models

**DOI:** 10.3389/fpsyg.2015.01097

**Published:** 2015-08-03

**Authors:** Sergio Morra, Erika Borella

**Affiliations:** ^1^DISFOR (Department of Education), Università di GenovaGenova, Italy; ^2^Department of General Psychology, Università di PadovaPadua, Italy

**Keywords:** working memory, training, task analysis, intellectual disabilities, transfer effects, standardized academic achievement outcomes, school outcomes

A first research wave on working memory (WM) training created an atmosphere of novelty and enthusiasm. Studies carried out with typical or atypical participants in different age ranges showed that training can improve WM efficiency, and the effects of training can transfer to IQ tests and other valued cognitive abilities (e.g., Klingberg et al., 2002; Jaeggi et al., 2008; Borella et al., 2013).

A second wave of research, in contrast, raised problems and criticisms, thus prompting a vein of skepticism. Issues brought to the fore concerned, for instance, adequacy of the control groups, the appropriate analysis of near and far transfer effects, and how to control for task-specific learning (e.g., Shipstead et al., 2012; Melby-Lervåg and Hulme, 2013; Redick et al., 2013).

A third research wave has started perhaps—and anyway, seems to be urgently needed. Current research should focus on clarifying which effects are obtained by which training programs. Training-related gains on tasks typical of daily life or on school outcomes (when children are considered), and their maintenance, should also be explored, as well as the role of individual differences, motivational and contextual factors, as discussed below. Most important, the theoretical framework of WM training research needs to be spelled out more clearly. (See also von Bastian and Oberauer, 2014).

The first wave yielded a wealth of potentially useful results, but most studies were rather atheoretical. Different research groups used different WM measures, such as complex span or *n*-back tasks. Was there a clear rationale for preferring one WM measure over another? Often, training involved a wide range of WM skills and executive functions; were any training components critical in producing the effects? In addition to specific methodological problems, we must consider a possible bias against publishing non-significant results, and potential interest conflicts inherent in carrying out research in collaboration with corporations that sell commercial WM training programs. These considerations point to a need to map the ground more clearly, with respect to which aspects of training produce which effects. However, this operation requires clear theoretical distinctions.

A simple metaphor—the “muscular metaphor”—seems to underlie many first-wave studies: doing WM gymnastics can strengthen the WM system, making it grow like a well-trained muscle; consequently, a larger WM can manage heavier workloads in complex cognitive tasks. Within this simple metaphorical framework, selecting one or another measure of WM is relatively unimportant. Moreover, using an unanalyzed mix of training components is no problem at all; the more varied the WM gymnastics, the more likely that it strengthens the system.

However, this metaphor is unlikely to explain adequately the WM-training benefits. After all, WM is not a muscle, and perhaps the effect of training is not simply to make it grow. Different WM theories might account differently for any training effects. Componential theories (Baddeley, 1986; Logie, 1995) assume that information is copied to domain-specific short-lived stores, coordinated by an executive system; if one assumes a componential theory, then it seems natural to ask whether a training program affects the domain-specific temporary stores or the central executive. Other theories, instead, assume that attentional resources are at the core of WM capacity—although different models, respectively emphasize activation resources (Pascual-Leone, 1970; Cowan, 2005), control processes (Engle et al., 1999), interference (Oberauer et al., 2012a), or time constraints on attention allocation (Barrouillet et al., 2009). These models view WM as the activated part of long term memory, and do not posit the existence of specialized temporary stores (although Cowan, 2005, does not exclude them in principle). Within these frameworks, one could investigate which attentional resources or control processes are affected by a training program. Improved WM efficiency (e.g., through strategies) should not be confused with expanded WM capacity. Improved performance in a trained WM task may not suffice to produce transfer effects; a specific effect in the trained task could be due to the use of particular strategies, or to a higher level of automation in the process(es) practiced in that task, but not to a greater WM capacity. On the other hand, here we suggest that there could be some transfer effects due to improved efficiency of the attentional processes that control resource allocation and use of WM.

Therefore, if we frame research questions within specific theories, choosing a WM measure is not just a matter of practical convenience; it carries various implications concerning “what” is trained and what changes occur in the cognitive system. Let us compare, for instance, complex span measures with *n*-back measures. To perform an *n*-back task, a person must maintain active representations of the previous *n* items and their serial order, encode the current item, compare it with the first of the memory list, make a decision, respond, drop the first item from the memory list, and update the list by including the last item and rearranging the order, so to continue with the next item—and all these operations must be performed under a certain time pressure. Improved *n*-back performance may reflect improvements in the efficiency or the speed of any or all of the foregoing operations, or in the control processes that manage the task, or in the use of any storage or attentional resources posited by a certain theory (e.g., to allocate activation energy to the relevant representations, or to resist interference from currently irrelevant items). To perform a complex span task, a person must encode one or more items of the processing task, perform the prescribed operations, encode an item of the memory task (possibly binding it with tags for relevance, order, etc.), keep the memory item(s) activated, and start over again with a cycle of the processing task, until recall of memory items is required. The demands of the processing task on WM capacity, control of interference, or speed of processing can vary across different complex span tasks. Improved performance in a complex span task may reflect improvements in any operation, control process, or structural component of the architecture of mind that is involved in the task.

Note that, although the differences between short-term memory tasks and complex span tasks are well-known, some WM training programs for individuals with intellectual disabilities (ID) combined a few WM tasks with other, mainly short-term memory tasks. It follows that it is important to reflect on what the tasks used for training WM involve. To understand improvement in WM measures, one must spell out a clear model of the processes that underlie that measure, and of those that are involved in the training program. It also seems appropriate to use more than one WM measure, so that one can compare measures that involve different processes, which are differently related to the training.

In some cases, detailed models were proposed for WM measures (e.g., Oberauer et al., 2012b for complex spans). Some theoretical approaches, in particular neo-Piagetian ones, emphasize the importance of detailed task analyses that consider the declarative and procedural information involved at each step of a task, as well as the processes that boost or hinder activation of the relevant cognitive units (Pascual-Leone and Johnson, 2011; Morra, 2015). This literature should not be ignored in studies on WM training.

Redick et al.'s (2013) findings provide remarkable food for thought in this line. Their participants, trained in a dual *n*-back task, improved dual n-back performance throughout the training, but showed no transfer to other measures of WM or intelligence. Such results show that a naïve “muscular metaphor” for WM training is clearly inadequate. We suggest that their training program affected task-specific processes, such as encoding the dual (visual-auditory) stimuli or their serial order. Comparing task-analytic models of successful and unsuccessful training studies could provide valuable insights on which types of training are most likely to be effective.

These reflections become crucial when WM training is intended for individuals with ID. Studies on WM training for individuals with ID found mainly near transfer effects, on tasks similar to the trained task. The goal of such programs is to improve the trainees' (normally children) general cognitive abilities, and the functional outcomes that rely on them, so achieving far transfer effects is crucially important. Training gains on untrained tasks were rarely reported, however. In addition, the training benefits in everyday abilities, skills related to academic outcome (in school-aged individuals), or in individual symptoms were examined surprisingly rarely (Melby-Lervåg and Hulme, 2013). When these aspects were considered, the results were contradictory, with benefits in daily life or symptoms being found in some studies, but not in others (Kirk et al., 2015). This inconsistency could be due to different training programs, or to the different measures used to assess far vis-à-vis near transfer effects of training, which are delicate methodological issues. But the picture remains equally cloudy even when we consider studies presenting the same program (i.e., Cogmed in the case of ADHD individuals), and assessing gains in the same cognitive processes (inhibition), or parents' ratings, symptoms, and academic achievement.

Standardized academic achievement tests could also shed light on the efficacy of WM training for children with ID, but they have rarely been considered. Partly because of great variability characterizing the profiles of children with ID, using such measures could enable us to assess the gains not only at group level but also for each individual. Thus, the utility of a training could be assessed from a more “clinical” standpoint. So far, however, the few studies that proposed WM training (in children with typical development) and used standardized measures to test its efficacy failed to demonstrate any effects, although there was evidence of improvement in other WM tasks (St. Clair-Thompson et al., 2010). Even though funding resources are not always sufficient to enable us to plan unimpeachable training studies (Gathercole et al., 2012), it would be important to schedule follow-up sessions to ascertain maintenance of WM training gains. Examining long-term effects becomes crucial in the case of individuals with ID. The lack of attention to these aspects in training individuals with ID is rather surprising considering how WM is involved in everyday cognitive and school activities. Improving these domains should be a high priority for individuals with ID.

Individual differences should be considered too when attempting to produce cognitive gains by training WM, because individuals with ID each have their own particular cognitive profile. WM training programs could be used in an effort to remedy cognitive impairments, and WM deficits are common in children with ID, but the severity of this impairment may be more pronounced in different processing domains. For instance, poor comprehenders have difficulties in verbal, but not in visuospatial WM tasks (see Carretti et al., 2009). Similarly, individuals with Down syndrome have an impaired verbal WM performance, with relatively more adequate performance in the visuospatial domain. On the other hand, children with nonverbal learning disabilities generally perform poorly on visuospatial, but not on verbal WM tasks. If we take the example of children with Attention Deficit Hyperactivity Disorder (ADHD), however, we find that most studies have focused on training visuospatial and phonological short-term tasks, instead of tasks that require a higher degree of executive control—in which actually they are more impaired, and which hinders more seriously their functional outcomes.

Baseline performance in WM tasks may also provide an indication of individual susceptibility to training. Given the great diversity of profiles seen in children with learning disabilities, it could also be that a minimal WM capacity is needed for any training to produce improvements, and that individuals with severe WM impairments will be unable to benefit from such programs. To the best of our knowledge, however, no WM training study conducted to date examined whether training effects vary across participants diagnosed with ADHD depending on its severity, and on any comorbidities.

Also, the important influence of motivational, emotional factors on WM and intellectual performance cannot be neglected. Recent studies suggest that compliance with a training program is of paramount importance to the improvements it can achieve (Jaeggi et al., 2014). Engagement with the program (training content) is therefore vital, but while typically-developing children can probably rely on their intrinsic motivation to complete a task, this may not be the case for individuals with ID or ADHD. Some training formats can sustain motivation and engagement more than others. Computer games that provide immediate feedback may be more effective than other training formats in motivating children with Down syndrome or ADHD, for instance. Motivation as a potential source of variability across studies was also examined only rarely, but it may have an impact—even on the control group. Although a determined effort is now being made to include active control groups, the proposed activities do not always include features that can sustain motivation, such as rewards (feedback), and they are not always as enjoyable or challenging as the activities used in the training program, so there is a risk of training gains being overestimated.

To sum up, WM training is a promising approach for sustaining individuals with ID. We have emphasized here, however, that while the focus on short-term cognitive benefits was justified in the very first WM training studies, the time has come for new training studies to clarify the theoretical framework, and concentrate on the task analysis of the training, and on the applied training outcomes and their maintenance.

## Conflict of interest statement

The authors declare that the research was conducted in the absence of any commercial or financial relationships that could be construed as a potential conflict of interest.
